# Subcutaneous Panniculitis-Like T-Cell Lymphoma (SPTL) in a Child with Spontaneous Resolution

**DOI:** 10.1155/2011/639240

**Published:** 2011-11-24

**Authors:** Achiléa L. Bittencourt, Maria das Graças Silva Vieira, Eny Guimarães Carvalho, Celeste Cunha, Iguaracyra Araujo

**Affiliations:** ^1^Department of Pathology, Complexo Hospitalar Universitário Prof. Edgard Santos, Federal University of Bahia, Salvador, BA, Brazil; ^2^Oncology Department, Pediatric Hospital Martagão Gesteira, Liga Baiana Contra a Mortalidade Infantil, Salvador, BA, Brazil

## Abstract

Subcutaneous panniculitis-like T-cell lymphomas (SPTLs) *α*/*β* are rare in childhood. The present report refers to a case of a 7-year-old male child presenting an extensive skin lesion that began when he was 5 years of age. Two biopsies were evaluated using the CD3, CD4, CD8, CD56, *β*F1, and TIA markers. A dense infiltrate of CD3+, CD4−, CD8+, CD56−, *β*F1+, and TIA+ pleomorphic lymphocytes was found in the subcutis. The previous biopsy showed cytophagic histiocytic panniculitis with a small focus on CD8+ and *β*F1+ malignant cells. The lesion regressed spontaneously. This case shows that prognosis may be excellent in SPTL (*α*/*β*). On the other hand, it also serves as an alert that a biopsy performed in an area of cytophagic panniculitis may lead to misdiagnosis.

## 1. Introduction

Subcutaneous panniculitis-like T-cell lymphoma (SPTL) was described in 1992 [1] as a new type of cutaneous T-cell lymphoma mimicking panniculitis. SPTL was subsequently included in the World Health Organization (WHO)'s classification of hematopoietic and lymphoid tissues as a tumor of either *α*/*β* or *γ*/*δ* cytotoxic T-cell phenotype [2]. However, in the latest WHO/European Organization for Research and Treatment of Cancer (EORTC) classification of primary cutaneous lymphomas, only SPTLs expressing an *α*/*β* phenotype were referred to as SPTL, whereas cases with a *γ*/*δ* phenotype were included in the group of peripheral T-cell lymphomas, unspecified, referred to as cutaneous *γ*/*δ* T-cell lymphomas (C*γ*/*δ*-TCLs) [3]. 

Recently, the EORTC Cutaneous Lymphoma Study Group has evaluated 63 cases of SPTL and 20 cases of C*γ*/*δ*-TCLs and showed clear differences between the *α*/*β* and *γ*/*δ* phenotypes. While the 5-year overall survival rate in cases of SPTL (*α*/*β*) is generally around 82%, with C*γ*/*δ*-TCL, the 5-year overall survival rate is 11% [4].

The objective of this paper was to describe an unusual case of SPTL (*α*/*β*) occurring in a child in whom involution was spontaneous.

## 2. Case Summary

**Figure 1:**
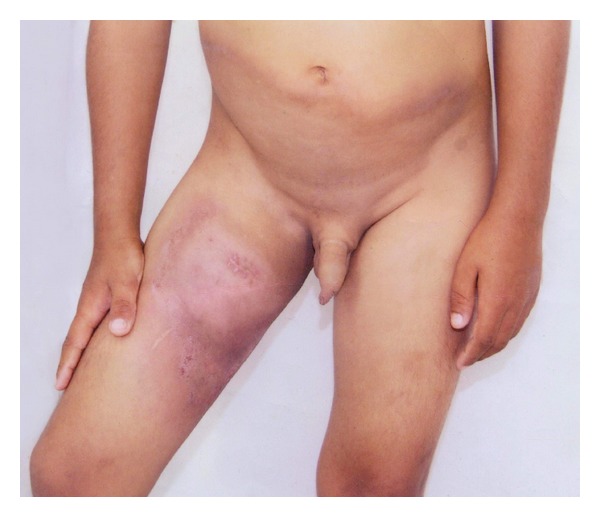
Extensive erythematous infiltrated plaque involving the abdomen and the right tight.

**Figure 2:**
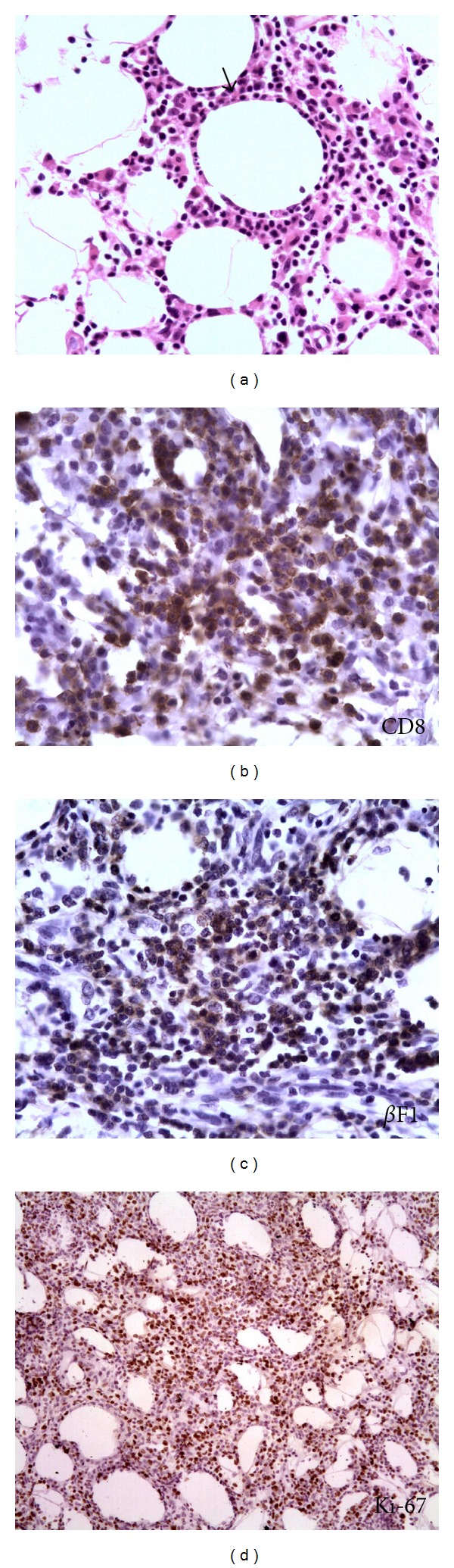
**(**a) Diffuse infiltration of small- and medium-sized pleomorphic lymphocytes is seen between the fat cells with rimming of the neoplastic cells around one individual fat cell (arrow) (H&E stain; original magnification ×320). (b) CD8 positivity of the neoplastic cells (original magnification ×400). (c) The majority of the cells presents the *αβ* phenotype (*β*F1, original magnification ×640). (d) A high proliferative index of the malignant cells is observed (Ki-67, original magnification ×125).

A 7-year-old male Brazilian child presented an extensive skin lesion that began when he was 5 years of age associated with constant fever varying from 38 to 39°C. The patient was HTLV-1 negative and HIV negative. Dermatological examination revealed diffuse erythema and infiltration of the skin involving the infraumbilical area and right inguinal region extending to the anterior and internal surfaces of the upper two-thirds of his right thigh (Figure 1). Routine laboratory tests revealed only mild anemia. The patient underwent one biopsy of the lesion which resulted in diagnoses of chronic panniculitis. Four months later, another biopsy was performed, and a diagnosis of SPTL (*α*/*β*) was made. The previous biopsy was reviewed. *Anatomopathological and immunohistochemical studies*. A dense and diffuse infiltrate of small and medium-sized lymphocytes with nuclear contour irregularity was found confined to the subcutaneous tissue. Rimming of the neoplastic cells surrounding individual fat cells was frequently found (Figure 2(a)). Many admixed histiocytes, small granulomas, and few foci with karyorrhectic debris were seen. Alcian blue staining revealed no mucin deposition. The neoplastic cells were CD2+, CD3+, CD4−, CD7+, CD8+ (Figure 2(b)), CD20−, CD30−, UCHL-1+, CD56−, CD79−, *β*F1+ (Figure 2(c)), and TIA+. Many CD68+ cells were seen amidst the neoplastic cells. The proliferative index (Ki-67) was 40% (Figure 2(d)). Diagnosis was SPTL (*α*/*β*). Review of the previous biopsy then revealed vacuolated histiocytes and epithelioid cells, with few lymphocytes in the fat lobules. Only a small, peripheral focus of interstitial infiltration of malignant lymphocytes CD3+, CD8+, and *β*F1+ was found. Taking the diagnosis of the latest biopsy into account, this was then considered to constitute a case of SPTL (*α*/*β*) admixed with a cytophagic histiocytic panniculitis. *Evolution*. In the seven months that it took to reach a conclusive diagnosis, during which no treatment was given, the patient underwent a progressive and accentuated reduction in infiltration. After the definitive diagnosis, he was submitted to the following staging investigations, all of which were normal, complete blood cell count, blood chemistry panel, chest radiography, computed tomography scan of the chest and upper and lower abdomen, and bone scintigraphy. There was no evidence of T-cell infiltrate or hemophagocytosis in the bone marrow smear or biopsy specimen. It was decided not to treat the patient. Examination of the patient four months later, showed only pigmentation of the skin at the site of the lesion. At the latest follow-up visit 20 months later, the patient continued in full remission.

## 3. Discussion

SPTL (*α*/*β*) affects a younger age group than C*γ*/*δ*-TCL, the median age of patients being 36 years of age. This is a rare type of lymphoma; however, many cases of SPTL (*α*/*β*) may be overlooked and may have been misdiagnosed as panniculitis [4]. Weenig et al. [5] described an adult case in which diagnosis was only possible after the eighth biopsy. In the case reported here, after reviewing the previous biopsy, a pattern of cytophagic histiocytic panniculitis was found, with one small, peripheral area containing atypical CD8+, CD56−, and *β*F1+ lymphocytes, confirming that this biopsy indeed corresponded to a case of SPTL (*α*/*β*).

It is impossible to be sure whether the observed panniculitis consists in fact of areas of involution of the tumor or whether it is rather a case of SPTL that has developed from cytophagic histiocytic panniculitis (CHP). According to Marzano et al. [6], CHP may progress to SPTL. However, considering that the lesion regressed progressively until its disappearance, it is more likely that CHP represents involution of the tumor.

In many studies reported in the literature that include children and adolescents, no differentiation was made between the *α*/*β* and *γ*/*δ* phenotypes [7–12]. On the other hand, in the few studies that included children and adolescents as well as adults and in which differentiation was made between these two phenotypes, the cases were not studied individually; therefore, it was impossible to obtain clinicopathological data on younger patients [13, 14]. There are few cases of SPTL (*α*/*β*) in children and adolescents of 18 years of age or less in which a differential diagnosis was performed with cutaneous *γ*/*δ* T-cell lymphomas by immunohistochemistry or by molecular biology [15–20]. These patients ranged from 6 months to 18 years of age. Clinically, the lesions appeared in the form of nodules or plaques. Four patients had systemic disease, two with hemophagocytic syndrome [15, 17]. In three cases, complete regression of the disease occurred following treatment, which consisted of chemotherapy in two cases [15, 16] and oral steroids in the remaining case [20]. Nevertheless, three deaths occurred, all in patients with systemic disease, which was associated in one case with hemophagocytic syndrome [15, 17]. In two cases, there was no reference to clinical characteristics, treatment, or outcome [18, 19]. Further studies involving a greater number of cases would be required to evaluate whether the disease behaves differently in children and adolescents compared to adults.

The present case was diagnosed as SPTL (*α*/*β*) rather than C*γ*/*δ*-TCL because of the clinical, anatomopathological, and immunohistochemical features present. The confinement of the infiltrate to the subcutaneous tissue, as observed in the present case, constitutes a feature that is helpful in the diagnosis of SPTL (*α*/*β*). Furthermore, unlike cases of C*γ*/*δ*-TCL, the malignant cells were CD4−, CD8+, CD56−, and *β*F1+ [4].

It is believed that some cases, originally diagnosed as lupus erythematosus profundus (LEP), may indeed have consisted of cases of SPTL (*α*/*β*) [4, 5]. Important clues to distinguish SPTL (*α*/*β*) from LED include epidermal involvement, interphase dermatitis, mucin deposition, and the presence of clusters of B cells or admixed plasma cells and reactive germinal centers [4, 21].

Considering that in the present case there was spontaneous involution of the disease, it is important to make a differential diagnosis with atypical lymphocytic lobular panniculitis (ALLP), a condition considered to represent a preneoplastic phase of SPTL (*α*/*β*), characterized by a subcutaneous lesion with a waxing and waning course. Some distinguishing morphological features are present in ALLP including the absence of a greater density of infiltration, a much lesser degree of cytologic lymphoid atypia, a low proliferative index, the presence of a significant number of CD4+ lymphocytes, and lack of *β*F1 expression [21]. In addition, interstitial mucin deposition is a characteristic feature of ALLP, and the striking dominance of CD8+ cells that typifies cases of SPTL (*α*/*β*) is absent [21]. According to Magro et al. (2007) [21], there are insufficient lymphoid atypia in ALLP for a diagnosis of lymphoma. Unlike the majority of SPTL (*α*/*β*) cases in the literature, complete spontaneous resolution of the lesion occurred in the present case with no further treatment. Spontaneous involution of this disease has been previously observed in only two of the 63 cases recently reviewed in the literature [4].

## 4. Conclusion

The current case shows that prognosis may be excellent with this form of lymphoma. On the other hand, it also serves as an alert that a biopsy performed in an area of cytophagic panniculitis may lead to misdiagnosis, hence, the importance of continued followup with repeated biopsies when SPTL (*α*/*β*) is suspected. A differential diagnosis between SPTL (*α*/*β*) and C*γ*/*δ*-TCL must be performed in all pediatric cases, and all cases of SPTL (*α*/*β*) should be published in order to provide pediatric oncologists with more complete and exact data on the morbidity and therapeutic management of this disease.
